# CYCLOPEp Builder: Facilitating cyclic peptide and nanotube research through a user-friendly web platform

**DOI:** 10.1016/j.csbj.2024.05.044

**Published:** 2024-06-02

**Authors:** Alfonso Cabezón, Fabián Suárez-Lestón, Juan R. Granja, Ángel Piñeiro, Rebeca Garcia-Fandino

**Affiliations:** a Department of Organic Chemistry, Center for Research in Biological Chemistry and Molecular Materials, University of Santiago de Compostela, CIQUS, Spain; b Soft Matter & Molecular Biophysics Group, Department of Applied Physics, Faculty of Physics, University of Santiago de Compostela, Spain; c MD.USE Innovations S.L., Edificio Emprendia, 15782 Santiago de Compostela, Spain

**Keywords:** Cyclic peptides, Self-assembling cyclic peptide nanotubes (SCPNs), Molecular, Builder, Supramolecular structures, Molecular dynamics simulations

## Abstract

The study of cyclic peptides (CPs) and self-assembling cyclic peptide nanotubes (SCPNs) is pivotal in advancing applications in diverse fields such as biomedicine, nanoelectronics, and catalysis. Recognizing the limitations in the experimental study of these molecules, this article introduces CYCLOPEp Builder, a comprehensive web-based application designed to facilitate the design, simulation, and visualization of CPs and SCPNs. The tool is engineered to generate molecular topologies, essential for conducting Molecular Dynamics simulations that span All-Atom to Coarse-Grain resolutions. CYCLOPEp Builder's user-friendly interface simplifies the complex process of molecular modeling, providing researchers with the ability to readily construct CPs and SCPNs. The platform is versatile, equipped with various force fields, and capable of producing structures ranging from individual CPs to complex SCPNs with different sequences, offering parallel and antiparallel orientations among them. By enhancing the capacity for detailed visualization of molecular assemblies, CYCLOPEp Builder improves the understanding of CP and SCPN molecular interactions. This tool is a step forward in democratizing access to sophisticated simulations, offering an invaluable resource to the scientific community engaged in the exploration of supramolecular structures. CYCLOPEp is accessible at http://cyclopep.com/

## 1 Introduction

In the quest to harness the intricate mechanisms of nature, scientists have ventured into the nano-realm, exploring the potential of cyclic peptides (CPs) as building blocks for supramolecular architectures [1], [2]. This exploration has unveiled CPs' exceptional capacity to fold and self-assemble into well-defined structures, such as Self-Assembled Cyclic Peptide Nanotubes (SCPNs), which hold great promise across diverse scientific fields [1], [3], [4], [5]. From biomedicine to nanoelectronics and catalysis, the applications of SCPNs are vast, driven by their unique sequence-dependent properties. The inception of SCPNs can be traced back to theoretical musings in 1974 [6], with the first synthetic steps taken in 1993 [7], employing alternating *D*,*L*-α-CPs to yield structures with significant potential as biomimetic transport systems or antimicrobial agents. However, studying the self-assembly of CPs into SCPNs has been limited by experimental constraints, propelling Molecular Dynamics (MD) simulations to the forefront [8], [9], [10], [11], [12], [13], [14]. MD simulations serve as a powerful tool to explore the self-assembly and dynamic behavior of SCPNs, providing valuable insights that are beyond the reach of direct experimental techniques. However, the challenge of generating initial three-dimensional structures for SCPNs presents a significant hurdle for these simulations. In contrast to proteins or other biomolecules whose structures are readily available in the Protein Data Bank [15], SCPNs do not have easily accessible 3D structures for reference. The task of constructing these structures with the molecular builders currently available is complex and challenging, presenting substantial obstacles not only for initiating MD simulations but also for their straightforward visualization and modeling.

Herein, we introduce CYCLOPEp Builder, a practical development in the design and simulation of CP sequences and SCPNs. This user-friendly platform is meticulously tailored to accommodate the needs of both the seasoned expert aiming to incorporate CPs into simulations and the inquisitive individual in search of a 3D structure of a particular CP or nanotube. CYCLOPEp Builder stands out as a multifunctional tool, providing an accessible gateway to the construction of CPs and SCPNs with correct topologies for simulation at both All-Atom (AA) and Coarse-Grain (CG) resolutions. Beyond facilitating MD simulations, CYCLOPEp Builder enhances the user's ability to visualize complex molecular assemblies, thus advancing the understanding of CP behaviors and interactions. It is a functional tool for both the simulation-driven investigation and the basic visualization of molecular constructs, offering an intuitive experience that bridges the gap between intricate molecular design and practical application. The platform democratizes access to advanced MD simulations and visualization, inviting a broad spectrum of the scientific community to engage with CPs in a context that is both accessible and scientifically robust.

## 2 Methods

**Fig. 1:**
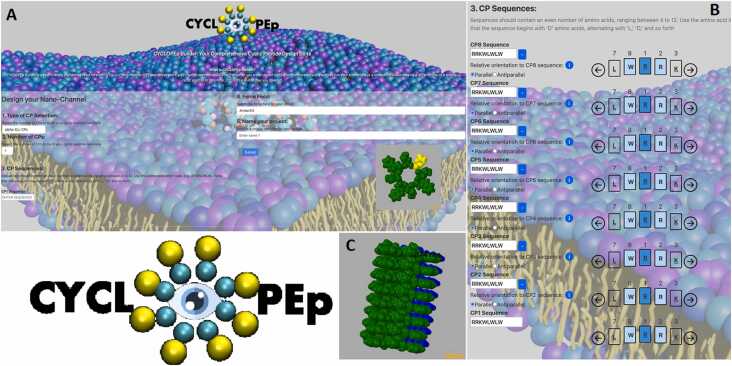
**A**. Main interface and logo of the CYCLOPEp application. **B**. Illustration of input sequences for a SCPN consisting of eight CPs with the sequence c-RRKWLWLW, in which the underlined letter corresponds to the D-aminoacid. **C**. Visualization of the resulting SCPN model generated by the application.

The CYCLOPEp Builder software functions as a web application, employing HTML for server-side operations and Javascript for client-side rendering of chemical structures and graphics, as shown in Fig. 1. For visualizing the molecular structures of CPs or SCPNs, JSmol [16] is utilized.

Users interact with the Python-based backend through a series of input fields within the web interface. The minimum requirements for generating a CP include the peptide sequence and a selected force field. If the user intends to construct a nanotube, all sequences and an additional input defining the peptides' orientation—parallel or antiparallel—are required.

It supports an array of force fields, at both AA and CG resolutions, including Amber03 [17], Amber94 [18], Amber96 [19], Amber99 [20], OPLS-AA [21], CHARMM36 [22], MARTINI 2.2 [23], MARTINI 2.2P [23], MARTINI 3.0 [24], and features the advanced MA(R/S)TINI [25] force field—a chirality-sensitive reparameterization of MARTINI—developed by our team to enhance the accuracy of CP representations.

The inputs provided by the user are automatically formatted by the web interface and conveyed to the Python script. The script constructs a tentative geometry for each CP, taking into account both the number of residues and the force field (AA or CG resolution), with the appropriate *D*/*L* alternation between adjacent amino acids. For consistency, the first written residue is a *D*-amino acid. The topology of the CP is adapted from the output of the *pdb2gmx* tool of GROMACS 2022.2 [26], [27] in the case of AA force fields, or generated through an algorithm developed within the script in the case of CG. The CP structure is then minimized, using a steepest descent algorithm (10,000 steps), executed in GROMACS 2022.2 [26], [27]. During this stage, specifically for AA force fields, various dihedral restraints are applied to maintain the backbone's open structure and the directional integrity of the N-H and C=O bonds, ensuring that the final structure is not only chemically accurate but also primed for schematic 3D modeling.

For the assembly into a SCPN, the script allows the CPs to rotate at precise angles, facilitating the formation of H-bonds between the CO and NH groups across adjacent CPs (Fig. 1B). This controlled rotation ensures that the peptides align correctly to establish the nanotube architecture. Once assembled, the nanotube undergoes a final minimization, maintaining the internal restraints from each CP's optimization to preserve the integrity and stability of the structure.

The final output from the script is a compressed file containing the PDB structures of the individual CPs and the complete nanotube, the sequence topologies for the selected force field, and a system index file. These files are formatted to be compatible with GROMACS [27], streamlining further simulation processes. The code uses Numpy [28] and SciPy [29] for numerical tasks, and MDAnalysis [30] for manipulating atomic and molecular data.

## 3 Overview of CYCLOPEp

CYCLOPEp Builder is a web-based platform designed for the facile generation and simulation set-up of CPs and SCPNs. It is designed to be user-friendly, with a straightforward interface that features the necessary text input areas and a dropdown menu at the top of the web page for easy navigation. Each input section is complemented by a brief explanation of the required information to guide the user smoothly through the process. Additional details, including a guide for using the website, the history of CPs, the comparison of AA and CG force fields for CP simulations, and a description of MA(R/S)TINI [25] are available at the bottom of the page, accessible via scrolling.

The data input process in CYCLOPEp Builder is multi-phased and is designed to be both comprehensive and accommodating:

1. Users begin by selecting the CP type from a range of α-, β-, γ-, δ-amino acids. Currently, the software is optimized and only available for the construction of α-*D*,*L*-CPs. In future updates, more CP types will be added to the selection options.
2. The number of CP sequences required for a design is specified via a dropdown selector, which in turn determines the number of sequence input fields to be displayed.
3. Each CP sequence is entered using the standard one-letter amino acid code. The software requires that each CP contains an even number of amino acids, allowing for sequences ranging from 4 to 12 residues. In the case of α-*D*,*L*-CPs, these should start with *D* amino acids and alternate with *L* amino acids.
4. If the design includes multiple CPs, the interface provides options to define the relative orientation—parallel or antiparallel— with respect to the previous subunit.
5. The user then selects the force field for the simulation from a dropdown menu. CYCLOPEp Builder supports a comprehensive range of force fields suitable for both AA (Amber03 [17], Amber94 [18], Amber96 [19], Amber99 [20], OPLS-AA [21] and CHARMM36 [22]) and CG (MARTINI 2.2 [23], MARTINI 2.2P [23], MARTINI 3.0 [24] and MA(R/S)TINI [25]). The choice of a force field in CYCLOPEp depends on the specific requirements of the simulation and the level of detail desired by the user. For instance, AA force fields provide detailed molecular interactions and are preferred for their high accuracy in MD simulations, making them suitable for studying the intricate behaviors and properties of CPs in environments where precise atomic details are necessary. On the other hand, CG force fields, such as MARTINI, offer faster computation times by simplifying the particles and interactions, which is beneficial for simulations that require large time scales or involve a large number of molecular entities. This simplification, while reducing computational load, might compromise some details but remains sufficient for studies focusing on larger-scale structural formations and dynamics.
6. Users can provide a distinctive name for their design or may use the automatically generated name that encapsulates the chosen force field and sequence information for easy identification.

After inputting all necessary data, the user can initiate the 'Submit' button, which triggers the backend Python script to run. The output is made available for download, and the nanotube.pdb file is rendered within the webpage through JSmol, enabling users to visualize their molecular system directly and interactively.

## 4 Conclusions

In this article, we presented CYCLOPEp Builder, a new tool developed for the design and simulation of CP sequences and SCPNs. The platform is designed with a focus on usability, aimed at supporting both experienced simulation professionals and those new to the field seeking detailed 3D models of CPs or nanotubes. CYCLOPEp Builder offers a versatile environment for building CPs and SCPNs, ensuring accurate topology for simulations at various levels of detail.

The software goes beyond merely enabling MD simulations; it also improves the visualization of molecular structures, contributing to a deeper comprehension of the behavior and interaction of CPs. As a resource for both in-depth research and foundational visualization, it provides a seamless link from complex molecular concepts to their tangible representations.

Importantly, the platform is not static; it is set to evolve, with planned updates to incorporate additional CP types and expand the force field options. This commitment to development ensures that CYCLOPEp Builder will continue to meet the advancing needs of both experienced researchers and newcomers to the field.

By making advanced MD simulations and visualization techniques more accessible, CYCLOPEp Builder extends an invitation to a wide range of researchers to explore the world of CPs and SCPNs. The platform is poised to be a staple in MD simulations research, serving as a catalyst for scientific engagement and progression in this specialized field.

## CRediT authorship contribution statement

**Juan R. Granja:** Writing – review & editing, Validation. **Fabián Suárez-Lestón:** Writing – review & editing, Writing – original draft, Visualization, Validation, Methodology, Investigation, Formal analysis. **Alfonso Cabezón:** Writing – review & editing, Writing – original draft, Visualization, Validation, Methodology, Investigation, Data curation. **Rebeca Garcia-Fandino:** Writing – review & editing, Writing – original draft, Visualization, Validation, Supervision, Project administration, Methodology, Investigation, Funding acquisition, Formal analysis, Data curation, Conceptualization. **Ángel Piñeiro:** Writing – review & editing, Writing – original draft, Visualization, Validation, Supervision, Methodology, Investigation, Funding acquisition, Formal analysis, Data curation, Conceptualization.

## Declaration of Competing Interest

The authors declare that they have no known competing financial interests or personal relationships that could have appeared to influence the work reported in this paper.
